# Supplementary material for: Ticks and Tick-Borne Pathogens in Popular Recreational Areas in Tallinn, Estonia: The Underestimated Risk of Tick-Borne Diseases
Source: Microorganisms. 2024 Sep 20;12(9):1918. doi: 10.3390/microorganisms12091918 (PMC11434170; doi:10.3390/microorganisms12091918)
Supplement: Supplementary file 1 [file microorganisms-12-01918-s001.zip › microorganisms-3200069-supplementary.pdf]

Table S1. PCR settings and primers used for the detection of tick-borne pathogens in tick nucleic acids extracts.

Target organism	Target gene (amplicon size, bp)	Method	Primer and probe names and 5´–3’ sequences	Cycling conditions	Reference
TBEV	3’ non-coding region (67 bp)	qRT-PCR	F-TBE1: GGG CGG TTC TTG TTC TCC<br>R-TBE1: ACA CAT CAC CTC CTT GTC AGA CT<br>TBE-probe-W: TGA GCC ACC ATC ACC CAG ACA CA	48 °C – 5 min, 95 °C – 30 sec, (95 °C – 3 sec, 60 °C – 30 sec) × 45	[18]
	E gene (465 bp)	RT-PCR	E frw1: GTT GTG TGG YTG ACY STG GA<br>E rev1: TCK GAK ACY TCY CTC CAC AC	65 °C – 5 min, on ice – 1 min, 25 °C – 5 min, 50 °C – 45 min, 70 °C – 15 min	[19]
		nested PCR	283F1: GAG AYC AGA GTG AYC GAG GCT GG<br>827R1: AGG TGG TAC TTG GTT CCM TCA AGT	(94 °C – 1 min, **57 °C** – 1 min, 72 °C – **2 min**) × 35	[13]
			349F2: GTC AAG GCG KCT TGT GAG GCA A<br>814R2: TTC CMT CAA TGT GYG CCA CAG G	(94 °C – 1 min, 60 °C – 1 min, 72 °C – 120 sec) × 30	
B. burgdorferi s.l.	*5S-23S intergenic spacer* (245-256 bp)	nested PCR	NC1: CCT GTT ATC ATT CCG AAC ACA G<br>NC2: TAC TCC ATT CGG TAA TCT TGG G	(94 °C – **30 sec**; 58 °C – **30 sec**; 72 °C – **1 min**) × 35	[21]
			NC3: CTG CGA GTT CGC GGG AGA<br>NC4: TCC TAG GCA TTC ACC ATA	(94 °C – **30 sec**; 52 °C – **30 sec**; 72 °C – **1 min**) × 30	
B. miyamotoi	*p66* (532 bp)	nested PCR	M1F: TTC TAT ATT TGG ACA CAT GTC<br>M2R: CAG ATT GTT TAG TTC TAA TCC G	(94 °C – **30 sec**, **52 °C**– **30 se**c, 72 °C – **1 min**) × 35	[20]
			M3F: CTA AAT TAT TAA ATC CAA AAT CG<br>M4R: GGA AAT GAG TAC CTA CAT ATG	(94 °C – **30 sec**, **49** °C – **30 sec**, 72 °C – **1 min**) × 35	
Anaplasmataceae	*16S rRNA* (1350 bp)	nested PCR	Ehr1: GAA CGA ACG CTG GCG GCA AGC<br>Ehr6: GAC CCA ACC TTA AAT GGC TGC	(94 °C – **30 sec**, **58 °C** – **30 sec**, 72 °C – **1 min**) × 35	[10]
			Ehr7: TAA CAC ATG CAA GTC GAA CG<br>Ehr8: CTT CGA GTT AAG CCA ATT CC	(94 °C – **30 sec**, **50 °C** – **30 sec**, 72 °C – **1 min**) × 35	
Rickettsia	*gltA* (74 bp)	qPCR	CS-F: TCG CAA ATG TTC ACG GTA CTT T<br>CS-R: TCG TGC ATT TCT TTC CAT TGT G	95 °C – 3min, (95 °C – 10 sec, 60 °C – 60 sec) × 40	[11]

		CS-P (probe): TGC AAT AGC AAG AAC CGT AGG CTG GAT G		
gltA (667 bp)	nested PCR	glt1: GAT TGC TTT ACT TAC GAC CC<br>glt2: TGC ATT TCT TTC CAT TGT GC	(94°C – **30 sec, 48°C** – **30 sec**, 72°C – **60 sec**) × 35	[23]
		glt3: TAT AGA CGG TGA TAA AGG AAT C<br>glt4: CAG AAC TAC CGA TTT CTT TAA GC	(94°C – **30 sec, 45.5°C** – **30 sec**, 72°C – **60 sec**) × 30	
sca4 (843 bp)	nested PCR	Sc4-1: ATG TCT CTG AAT TAA GCA ATG C<br>Rj2837r: CCT GAT ACT ACC CTT ACA TC	(94°C – 60 sec, 52°C – 60 sec, 72°C – 2 min) × 35;	
		sc4-3: AAT TAT TAG GCT CTG TAT TAA AGA<br>sc4-4: GAA AGG ATA GCA CGA AAA GTA	(94°C – 60 sec, 50°C – 60 sec, 72°C – 2 min) × 30	
ompB (769 bp)	PCR	120-2788F: AAA CRA TAA TCA AGG TAC TGT<br>120-3599R: ACY STG GAR AGT GTG GTG AC	(95°C – 30 sec, 51°C – 30 sec, 72°C – 60 sec) × 35	[20]

Cycling conditions modifications are indicated bold.

reaction mix contents:

qRT-PCR: final volume of 20 uL, containing Quanta qScript One-Step Fast qRT-PCR kit with low ROX (Quantabio, Beverly, MA, USA), forward and reverse primers at final concentration of 900 nM each, 200 nM of probe and 5 uL of RNA/positive/negative control.

RT-PCR: SuperScript III Reverse Transcriptase kit (ThermoFisher Scientific, USA)

qPCR: final volume of 20 uL, containing Takyon Low ROX Probe qPCR Mastermix (Kaneka Eurogentec, Belgium, EU) with each primer concentration at 900 nM and probe at 200 nM

PCR and nested PCR: final volume of 25 µl, containing 10X DreamTaq PCR buffer (ThermoFisher Scientific, USA), per 0.8 mM of each dNTPs, 0.5 mM of each forward and reverse primers, per 1.5 mM $MgCl_2$ for outer and 1 mM $MgCl_2$ for inner reaction and 1 U of DreamTaq DNA Polymerase (ThermoFisher Scientific, USA) and 5 µl of cDNA, DNA or PCR reaction product
